# Phylogeography and transmission of *Mycobacterium tuberculosis* spanning prisons and surrounding communities in Paraguay

**DOI:** 10.1038/s41467-023-35813-9

**Published:** 2023-01-19

**Authors:** Gladys Estigarribia Sanabria, Guillermo Sequera, Sarita Aguirre, Julieta Méndez, Paulo César Pereira dos Santos, Natalie Weiler Gustafson, Margarita Godoy, Analía Ortiz, Cynthia Cespedes, Gloria Martínez, Alberto L. García-Basteiro, Jason R. Andrews, Julio Croda, Katharine S. Walter

**Affiliations:** 1 Instituto Regional de Investigación en Salud, Caaguazú, Paraguay; 2 Instituto de Salud Global de Barcelona (ISGLOBAL), Barcelona, Spain; 3 Programa Nacional de Control de la Tuberculosis, Ministerio de Salud Pública y Bienestar Social (MSPyBS), Asunción, Paraguay; 4 Postgraduate Program in Infectious and Parasitic Diseases, Federal University of Mato Grosso do Sul, Mato Grosso do Sul, Brazil; 5 Laboratorio Central de Salud Pública (LCSP), Ministerio de Salud Publica y Bienestar Social (MSPyBS), Asunción, Paraguay; 6 Centro de Investigação em Saude de Manhiça (CISM), Maputo, Mozambique; 7 Centro de Investigación Biomédica en Red de Enfermedades Infecciosas (CIBERINFEC), Barcelona, Spain; 8 Division of Infectious Diseases and Geographic Medicine, Stanford University School of Medicine, Stanford, CA USA; 9 Federal University of Mato Grosso do Sul - UFMS, Campo Grande, MS Brazil; 10 Oswaldo Cruz Foundation Mato Grosso do Sul, Mato Grosso do Sul, Brazil; 11 Department of Epidemiology of Microbial Diseases, Yale School of Public Health, New Haven, USA; 12 Division of Epidemiology, University of Utah, Salt Lake City, UT 84105 USA

**Keywords:** Tuberculosis, Molecular evolution, Phylogenetics

## Abstract

Recent rises in incident tuberculosis (TB) cases in Paraguay and the increasing concentration of TB within prisons highlight the urgency of targeting strategies to interrupt transmission and prevent new infections. However, whether specific cities or carceral institutions play a disproportionate role in transmission remains unknown. We conducted prospective genomic surveillance, sequencing 471 *Mycobacterium tuberculosis* complex genomes, from inside and outside prisons in Paraguay’s two largest urban areas, Asunción and Ciudad del Este, from 2016 to 2021. We found genomic evidence of frequent recent transmission within prisons and transmission linkages spanning prisons and surrounding populations. We identified a signal of frequent *M. tuberculosis* spread between urban areas and marked recent population size expansion of the three largest genomic transmission clusters. Together, our findings highlight the urgency of strengthening TB control programs to reduce transmission risk within prisons in Paraguay, where incidence was 70 times that outside prisons in 2021.

## Abstract (web summary)

To role that carceral institutions play in *Mycobacterium tuberculosis* transmission remains somewhat unknown. Authors perform a prospective genomic surveillance study, to assess transmission dynamics in prisons and surrounding communities in Paraguay.

## Introduction

Despite significant tuberculosis (TB) control efforts, the incidence rate of TB has declined only slowly in the World Health Organization Region of the Americas, and, alarmingly, has stagnated since 2014^1^. The COVID-19 pandemic disrupted access to healthcare—including critical TB diagnostic and treatment programs—compounding the burden of TB and reversing decades of progress in TB control^1^.

New approaches to limit transmission are urgently needed in Paraguay, where TB control is chronically underfunded^1^ and where TB incidence was 48 (41–56) per 100,000 people in 2020, higher than the mean incidence rate across the region^1^. More than a quarter of the country’s population lives below the national poverty line^2^ and are at heightened risk of TB infection and mortality. Further, recent dramatic increases in incarceration^3,4^ put a rapidly growing population at high risk of infection and disease^5–7^. To guide interventions in Paraguay, there is a critical need to identify the populations at greatest risk of infection and locations and institutions where transmission most frequently occurs^8^.

Whole genome sequencing of the *Mycobacterium tuberculosis* complex has been powerfully applied to characterize recent transmission dynamics. Genomic approaches have dated introductions of *M. tuberculosis* and reconstructed patterns of historic spread across Central and South America^9,10^, estimated the contribution of recent transmission to incident TB cases^11^, reconstructed the emergence of resistance-associated mutations^12^, and inferred likely individual-level transmission events^13^. In Brazil^14^ and Georgia^13^, for example, genomic approaches identified frequent transmission within prisons as well as evidence of spillover from prisons to surrounding communities. A single *M. tuberculosis* molecular study from Paraguay^15^ on strains collected in 2003 reported that *M. tuberculosis* families found across South America, including the Latin-American (LAM; sub-lineage 4.3) and Haarlem (4.1.2.1) were also common in Paraguay^15,16^.

Genomic approaches have not been applied to address major gaps in our understanding of TB transmission in Paraguay. First, the conditions of incarceration put people at high risk of many infectious diseases, and globally, over the past twenty years, the incarcerated population in Central and South America has grown by 206%, the greatest increase in the world^4^. Escalating incarceration rates have been paralleled by an increasing concentration of notified TB among incarcerated individuals^6^. Yet the role of prison environments on TB transmission both inside and beyond prisons, as sources of broader infection, has not yet been described in Paraguay. Second, while incidence of TB is heterogeneous across the country^15^, it remains unknown whether specific cities or regions function as hotspots, fueling transmission elsewhere. Finally, due to limited surveillance infrastructure, the prevalence of drug-resistance and multi-drug-resistance has not yet been systematically measured^17–19^. Only 56% of bacteriologically confirmed new cases of pulmonary TB were tested for rifampicin resistance in 2020^1^.

To characterize transmission dynamics and circulating diversity of *M. tuberculosis* complex strains in Paraguay, we conducted prospective genomic surveillance across the country from 2016 to 2021, including surveillance within and outside prisons, generating a genomic resource for continued surveillance in Paraguay. We estimated the role of likely recent *M. tuberculosis* transmission within prisons, the relatedness of prison and community transmission, and the frequent movement of *M. tuberculosis* between Paraguay’s urban centers.

## Results

### Population-based genomic surveillance

**Fig. 1:**
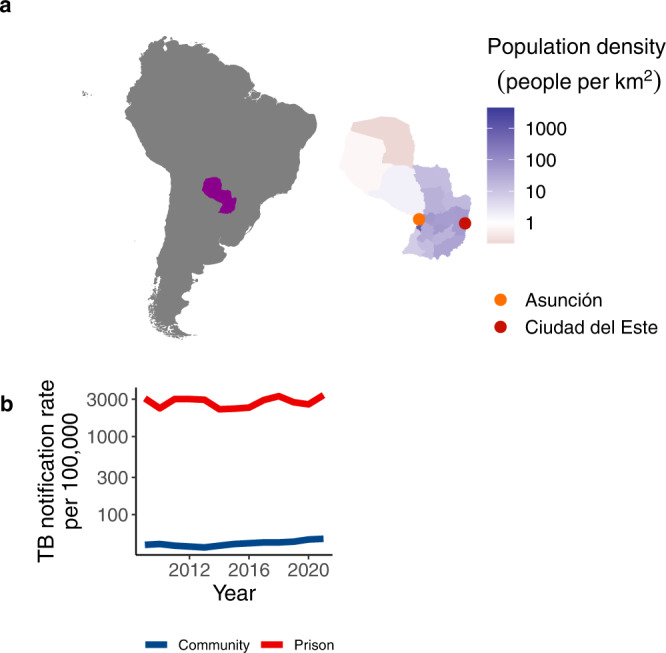
Genomic surveillance within and outside prisons in Paraguay’s urban centers. **a** Map of South America, with Paraguay highlighted, and as an inset. Paraguay’s departments are colored by population size density and points indicate the two largest urban centers in Paraguay, where we conducted focused genomic surveillance. **b** Notification rate of TB per 100,000 people in prisons (red) and in the general population (blue) from 2009 to 2020. Source data are provided as a Source Data file.

From 2016 to 2021, 16,734 TB cases were notified in Paraguay, with the majority of cases (60%; 10,095/16,734) occurring in the urban departments Central and Distrito Capital (which together comprise Asunción) and Alto Paraná (Ciudad del Este), where we conducted prospective genomic surveillance (Fig. 1a, Fig. S1). In 2021, the TB notification rate was 70 times higher in prisons than outside (3378 cases per 100,000 in prisons/49 cases per 100,000 in the general population) (Fig. 1b). Therefore, we focused genomic surveillance in the two largest prisons in the country, Tacumbú Prison and the Prison of Ciudad del Este, which together hold 36% (4950/13,821) of Paraguay’s incarcerated population, notification rates are 2000 and 3500 per 100,000 people, respectively.

Of the 7780 TB cases notified in Asunción during the study period, 781 (10%) occurred among incarcerated individuals (Fig. S1). 64% (503/781) of these were culture-positive and, of these, we sequenced 21% (107/503). 33% (2306/6999) of non-incarcerated individuals with TB in Asunción were culture-positive and of these, we sequenced 7% (172/2,306). Of the 2,315 TB cases notified in Ciudad del Este during the study period, 422 (18%) occurred among incarcerated individuals (Fig. S1). 64% (269/422) of these were culture-positive and, of these, we sequenced 20% (55/269). 31% (578/1893) of non-incarcerated individuals with TB in Ciudad del Este were culture-positive and of these, we sequenced 27% (158/578) (Fig. S1).

Whole genome sequences (WGS) for a total of 532 isolates met our coverage and quality criteria (Methods), including 488 from unique TB notifications. Of the samples passing filters, 158 were from individuals diagnosed with TB while in prison and 330 were from people diagnosed in the community. TB isolates were collected in Asunción (274/488) and in Ciudad del Este (214/488). We excluded 17 isolates with evidence of mixed infection with more than one sub-lineage detected, resulting in 471 *M. tuberculosis* isolates for following analyses.

### Genotypic resistance

**Fig. 2:**
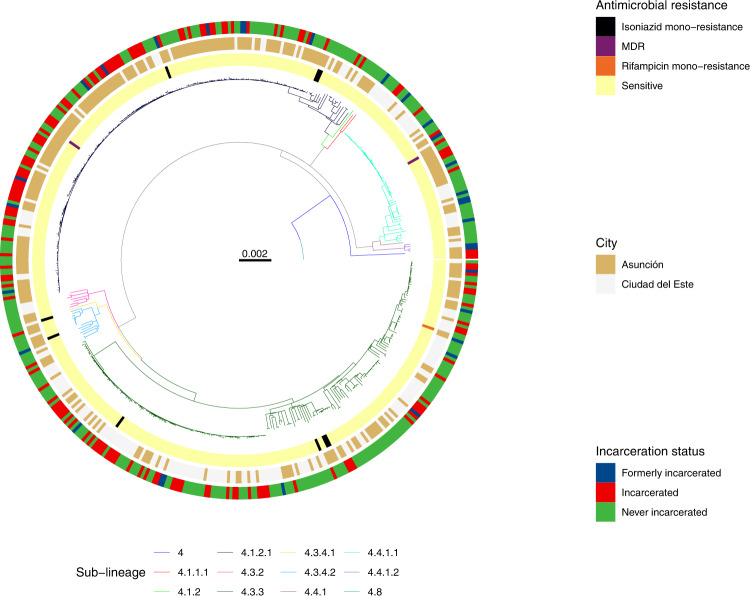
*M. tuberculosis* isolates from incarcerated and non-incarcerated people are closely related across Paraguay. A maximum likelihood phylogeny of 471 tuberculosis isolates from Lineage 4 inferred from a multiple sequence alignment of 9966 SNPs and rooted on three sub-lineage 4.8 samples from this study. Branch lengths are in units of substitutions per site. Branches are colored by sub-lineage. From the inside, rings are colored by antimicrobial resistance; city of sampling; and incarceration status at the time of TB notification. Other isoniazid includes isoniazid mono-resistant isolates without an *ahpC* promoter mutation. Rifampicin indicates rifampicin mono-resistant isolates. Other resistance includes isolates with mutations associated with resistance to pyrazinamide, streptomycin, or fluoroquinolones and not isoniazid or rifampicin. Source data are provided as a Source Data file.

The majority, 96% (454/471) of sampled *M. tuberculosis*, were drug-sensitive; 3% (15/471) were resistant to at least one drug; and 0.42% (2/471) were multi-drug resistant, resistant to both isoniazid and rifampin. Resistance was not associated with sub-lineage (*X*^2^ (11) = 7.7, *p* = 0.74). We identified three unique isoniazid resistance-conferring mutations on the genes *fabG1*, *katG*, or both among the 10 isolates with any isoniazid resistance; the three rifampicin-conferring mutations in *rpoB* (two on multi-drug resistant isolates) were unique (Fig. 2).

### Stable genomic diversity of *M. tuberculosis* in Paraguay

After excluding mixed infections, all *M. tuberculosis* isolates were strains from *M. tuberculosis* lineage 4. A single mixed lineage infection was co-infected with strains from both lineages 1 and 4. Samples predominantly fell into four sub-lineages: 4.3.3/LAM (42.5%; 200/471), 4.1.2 /Haarlem (38.2%; 180/471), 4.4.1/S (12.3%; 58/471), and 4.3.4/LAM (3.2%; 15/471) (Fig. 2). The distribution of strains representing these sublineages was stable and did not change significantly from a collection of 173 *M. tuberculosis* isolates collected in 2003^15^ (Fig. S1).

### Recent expansion of *M. tuberculosis* transmission clusters

We next explored evidence of recent *M. tuberculosis* transmission in Paraguay. As seen in a maximum likelihood phylogeny (Fig. 2), sampled *M. tuberculosis* diversity was dominated by several highly related clones. Seventy-eight percent (369/471) of all isolates fell within 26 genomic clusters (each including 2 to 159 isolates) defined by a 12-SNP threshold^20^, suggesting TB notifications were often attributable to recent transmission.

**Fig. 3:**
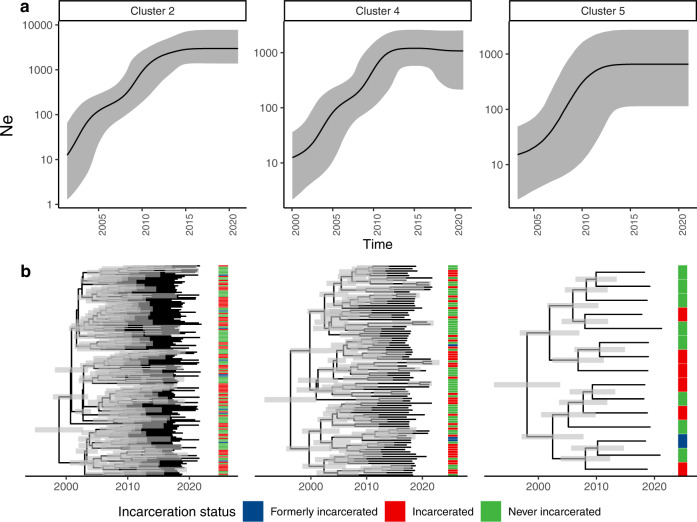
Genomic transmission clusters spanning prisons and neighboring communities have recently expanded. **a** Effective population size (*Ne*) estimates for the three largest genomic clusters in our sample over time. Black lines indicate *Ne* inferred in a Bayesian Skyline Coalescent model and grey shading indicates 95% high posterior density estimates. **b** Median clade credibility trees inferred from the Bayesian Skyline Coalescent model. Branch lengths are in years and grey bars indicate 95% high posterior density estimates of node date. The heatmap to the right of the phylogeny indicates patient incarceration status at the time of TB notification. Source data are provided as a Source Data file.

We reconstructed population size dynamics of the three largest genomic clusters—which comprised 56% (264/471) of our sample—with a Bayesian coalescent population size model. The three largest genomic clusters (including 159, 91, and 15 samples) increased in effective population size by 200, 90, and 40-fold, respectively. Cluster growth was relatively recent, with cluster most recent common ancestors (MRCA) occurring in 1998 (95% HDI: 1994–2001), 1996 (95% HDI: 1991–2000), and 1998 (95% HDI: 1992–2003) respectively, to 2021, when the most recent samples were collected (Fig. 3). All three clusters included isolates from individuals notified with TB during incarceration and individuals with no incarceration history.

We found no evidence that genomic loci were associated with successful genomic clusters, which we defined as the clusters containing more than 15 *M. tuberculosis* isolates, using a bacterial GWAS approach which controls for clonality and strong population structure^21^. We similarly found no association between genomic loci and clustered phenotype when we examined membership in a cluster with 10 or more isolates or 2 or more isolates.

### *M. tuberculosis* genomic clusters span prisons and the general population

In a maximum likelihood phylogeny (Fig. 2), M. *tuberculosis* isolates sampled from incarcerated and non-incarcerated people are distributed across the tree and did not form distinct clades, indicating a recent shared evolutionary history of isolates sampled from prisons and the community. However, sub-lineage was associated with incarceration status (*χ*^2^(22) = 52.3, *ρ* < 0.001), with strains from sub-lineage 4.1.2.1 more frequently infecting people with a history of incarceration (46.1%; 83/180) compared to individuals with no incarceration history (33.0%; 96/291; *p* = 0.006).

Isolates from incarcerated people were more frequently clustered (92.6%, 138/149), than those from formerly incarcerated (71.0%, 22/31, *χ*^2^(1) = 10.1, *ρ* = 0.001) or never incarcerated people (71.8%, 209/291; *χ*^2^(1) = 24.3, *p* < 0.001), likely reflecting more recent transmission within prisons. With a stricter threshold of 5 SNPs, 45.4% (214/471) of all isolates in genomic transmission clusters. With this threshold, isolates from incarcerated individuals were again more frequently clustered (58.3%; 87/149) than those from those formerly incarcerated (45.2%; 14/31), though not significantly so (*χ*^2^(1) = 1.3, *ρ* = 0.25), and isolates from incarcerated individuals were more frequently clustered than those from never incarcerated individuals (38.8%; 113/291; *χ*^2^(1) = 24.3, *ρ* < 0.001).

We predicted that if prison and community-associated epidemics were distinct, isolates from the community would be most closely related to and cluster with other isolates from the community and vice versa. Approximately half (48.0%; 12/25) of genomic clusters, including people with no incarceration history also included individuals with a recent history of incarceration. The consequence is that 85.2% (178/209) of individuals with evidence of recent transmission and no recent incarceration were within transmission clusters, including individuals with prior incarceration.

We additionally quantified *M. tuberculosis* recent transmission with time-scaled haplotype diversity, a measure of the centrality of a single tip isolate to all other isolates on the tree^22^. Individuals who were incarcerated at the time of TB notification had a higher time-scaled haplotype index for a short epidemic timescale (median: 0.59, IQR: 0.24–0.72) than did formerly (median: 0.18, IQR: −0.37–0.71; t(36) = 1.7, *p* = 0.03) or never incarcerated individuals (median: 0.20, IQR: −0.71–0.66; t(360) = 5.9, *p* < 0.001) (Fig. S3). This finding was consistent across epidemic timescales considered (Fig. S3). After adjusting for population structure, we found that incarceration status was significantly associated with time-scaled haplotype diversity (one-way ANOVA: F(285) = 85, *p* < 0.001), evidence that the association was independent of TB lineage.

### Geographic structure despite frequent migration across *M. tuberculosis* sub-lineages

**Fig. 4:**
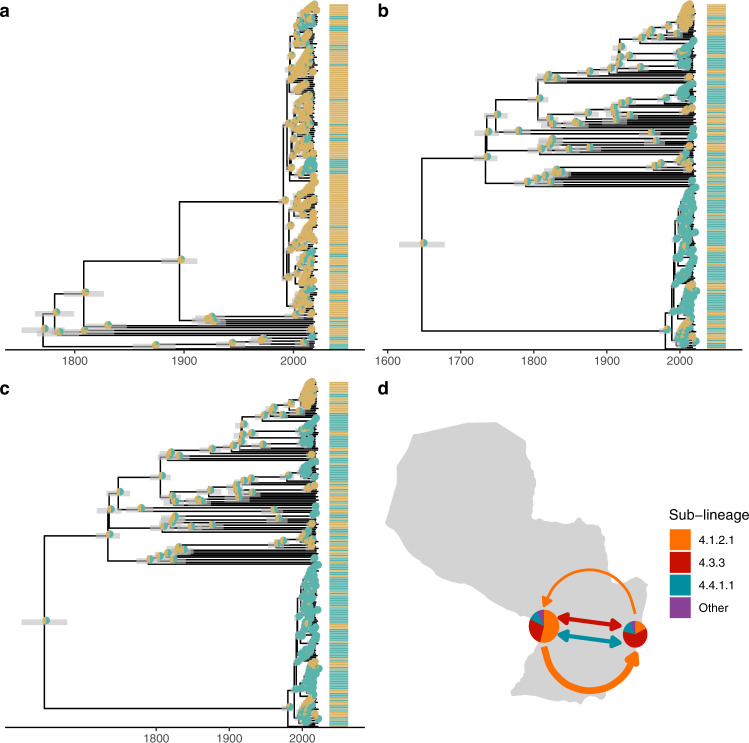
Frequent gene flow of *M. tuberculosis* connects Paraguay’s major urban centers. **a**–**c** We used discrete ancestral state reconstruction to reconstruct migration between the two cities for the three dominant sub-lineages in our sample; lineages 4.1.2.1, 4.3.3, and 4.4.1.1. Bayesian maximum clade credibility trees of samples in the three dominant sub-lineages with tip points colored by city of sampling and pie charts at nodes indicating the inferred ancestral location. Branch lengths are in years and grey bars indicate 95% high posterior density estimates of node date. A model of asymmetric rates of movement between the two cities was supported for sub-lineages 4.1.2.1 a model of symmetric rates of movement was supported for 4.3.3 and 4.4.1.1. **d** Map of Paraguay with pie charts indicating the genomic diversity sampled in Asunción (at Paraguay’s western border) and Ciudad del Este (eastern border). Arrows are colored by sub-lineage and are weighted by the relative rate of migration between cities. Bi-directional arrows indicate equal rates of migration in each direction. Source data are provided as a Source Data file.

We found pattern of moderate geographic structure in sampled *M. tuberculosis* (Fig. 4), with strains from sub-lineage 4.1.2.1 dominant in Asunción (54.1%, 142/262 samples) and strains from sub-lineage 4.3.3 dominant in Ciudad del Este (60.8%, 127/209) (*χ*2(2) = 72, *ρ* < 0.001) (Fig. 4). While we observed geographically distinct patterns of *M. tuberculosis* diversity in Asunción and Ciudad del Este, reconstruction of the ancestral locations for the three most prevalent sub-lineages revealed frequent movement of *M. tuberculosis* (Fig. 4).

To test whether Asunción and Ciudad del Este served as sources for *M. tuberculosis*, exporting infection elsewhere, we compared rates of arrival and export of each sub-lineage. Sub-lineage 4.1.2.1 moved more frequently Asunción to Ciudad del Este (mean: 75 transitions) compared to vice versa (mean: 70 transitions), and a model for asymmetric rates was supported (*χ*^2^(2) = 4.1, *ρ* = 0.04) (Fig. 4). Both sub-lineages 4.4.1.1 (with the prevalent *ahpC* mutation) (*χ*^2^(2) = 0.16, *ρ* = 0.69) and 4.3.3 (*χ*^2^(2) = 0.56, *ρ* = 0.46) had similar rates of migration to and from Ciudad del Este to Asunción. Despite the geographic structure observed, there was not a sufficient signal to infer a likely geographic source for any of the dominant sub-lineages.

### Emergence of a putative resistance-associated *ahpC* promoter mutation

**Fig. 5:**
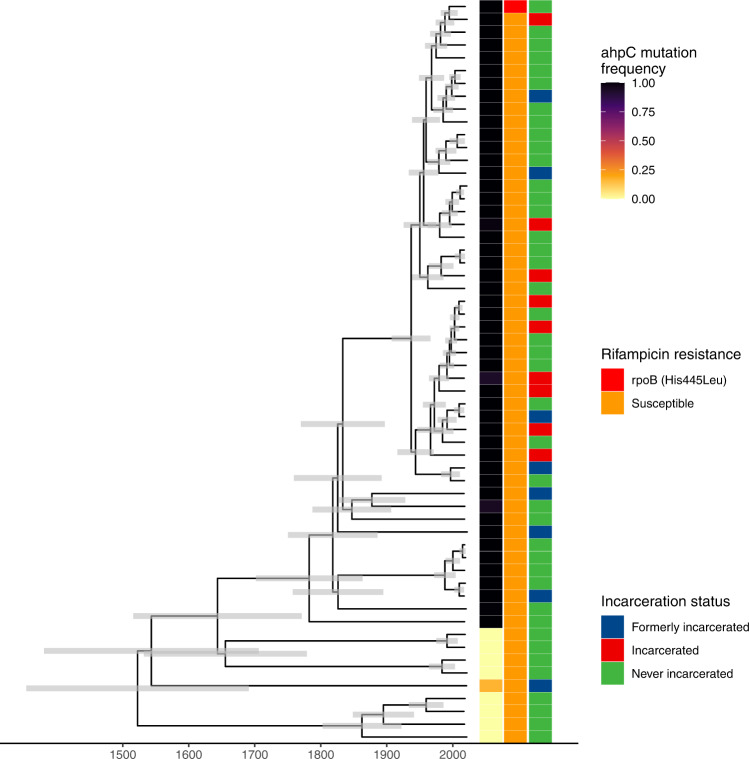
Emergence of a putative resistance-associated *ahpC* promoter mutation. Time-scaled Bayesian maximum clade credibility tree for 58 samples in sublineage 4.4.1.1. Branch lengths are in years and grey bars indicate 95% high posterior density estimates of node date. The heatmap to the right of the phylogeny indicates patient incarceration status at the time of TB notification, *ahpC* mutation frequency within an individual’s infection, and the occurrence of a rifampicin resistance-conferring mutation (*rpoB* His445Leu). Source data are provided as a Source Data file.

Eleven percent of samples (50/471) shared a mutation in *ahpC* promoter (G > A, 74 bases upstream of the 5’ start codon), previously considered a location for compensatory mutations co-occurring with *katG* mutations in isoniazid-resistant isolates^23,24^. While *ahpC* promoter mutations are not included as an independent resistance-conferring mutation in the WHO resistance catalogue^23^, in our collection, *ahpC* mutations occurred on otherwise susceptible genomic background within sub-lineage 4.4.1.1. The *ahpC* mutation occurred in a monophyletic clade of 49 samples in sublineage 4.4.1.1 (Fig. 5), which shared a most recent common ancestor in 1903 (95% HDI: 1888–1916), likely reflecting a single emergence event. Among the basal group of nine samples without a fixed *ahpC* promoter locus (*ahpC*−74) mutation, one sample was polymorphic, with 16% (13/79) of reads representing the *ahpC* mutation. Among the samples sharing the *ahpC* mutation, a single isolate had a co-occurring rifampicin resistance-conferring mutation in *rpoB* (His445Leu) (Fig. 5).

We tested whether the success of the *ahpC* mutation in the absence of a *katG* mutation (i.e. outside of a compensatory context) we observed in Paraguay could be explained by an increase in associated transmissibility. The *ahpC* mutation was not associated with an increased time-scaled haplotype density (*ahpC* mutants, median 0.19, IQR: 0.09–0.22; *ahpC* non-mutants, median: 0.56, IQR: −0.44–0.71, *p* = 0.93). Further, individuals with an incarceration history (currently or formerly incarcerated) were no more likely to be infected with a *M. tuberculosis* isolate with the *ahpC* mutation than were individuals with no incarceration history (*χ*^2^(1) = 0.25, *ρ* = 0.62).

## Discussion

We generated, to our knowledge, the first genomic portrait of circulating *M. tuberculosis* diversity and transmission dynamics to directly inform Paraguay’s national TB control program priorities. We found the majority of TB cases included in our study were likely attributable to recent transmission and identified three dominant clones, which dramatically expanded over the past twenty years and spanned prisons and surrounding communities. Overall, we found a pattern of close genomic relatedness between *M. tuberculosis* sampled within and outside prisons. While *M. tuberculosis* is geographically structured in Paraguay, we identified a signal of continuous movement of *M. tuberculosis* between Paraguay’s major urban centers.

We found that most sampled infections were likely attributable to recent transmission rather than long-distance migration or activation of latent disease, similar to what has been reported in other medium-incidence countries^14^. Consistent with expectations that clustering rates may correlate with incidence, when applying a 5-SNP threshold, we found that isolates from Paraguay were more frequently clustered (45%) than those from a low-incidence setting in Spain (23%) and less frequently clustered than in a high-incidence setting in Mozambique (58%). Interestingly, we found a higher rates of clustering compared to what was reported in Malawi (36%), a high-incidence setting^11^. This could reflect the shorter, one year sampling timeframe of the Malawi study^11^, resulting in different genomic sampling rates, the use of different genomic sequencing pipelines, or true differences in transmission in the sampled population.

Paraguay’s incarceration rate has dramatically increased, from 60 per 100,000 people in 2000 to 194 per 100,000 in 2020^3,4,6^. More than seventy percent of the incarcerated population are pre-trial detainees, the highest proportion in South America^3^. The unhealthy conditions of prison environments put people at heightened risk of disease and mortality; this risk translates into an increasing concentration of TB within prisons, with 18% (537/2593) of notified TB cases in Paraguay occurring among incarcerated individuals in 2020^6^. Paraguay’s TB Control Program has worked in prisons since 2004 to provide trainings for healthcare providers and all diagnostic and treatment supplies, including laboratory capacity for microbiological testing in four prisons.

Our findings highlight the critical need to expand and strengthen existing programs to detect and treat TB early and to expand awareness and knowledge of the risks associated with prison environments. Isolates sampled from prisons were more frequently found in genomic transmission clusters and had a higher time-scaled haplotype density than did isolates from outside prisons, phylogenetic evidence that recent transmission was more frequent in prisons than in communities outside prisons. Further, *M. tuberculosis* sampled from prisons and the community were closely evolutionarily related and the majority of putative transmission clusters, including individuals who were never incarcerated also included people who had a recent incarceration history, indicating that reducing transmission risk within prisons is an urgent public health priority with consequences both within and outside prisons.

While rates of drug resistance were relatively low, we found several phylogenetically unique mutations associated with both isoniazid and rifampicin resistance. These unique mutations could reflect either the de novo acquisition of a resistance mutation or the importation of a resistance mutation from outside Paraguay. Regardless, there is a critical need for expanding drug-susceptibility testing, including both rapid testing for rifampicin resistance in addition to isoniazid monoresistance are critical to ensure patients are put on correct treatment courses and to reduce the risk of further resistance acquisition^18,19^.

The emergence of an independent *ahpC* mutation within a single sublineage opens questions about its phenotypic consequences. Previous studies in laboratory strains have reported that *ahpC* mutations are compensatory in the context of *katG* isoniazid resistance-conferring mutations, by recovering the bacterium’s ability to detoxify organic peroxides, but did not find measurable isoniazid resistance conferred by independent *ahpC* mutations^24^. Genome-wide association studies of clinical *M. tuberculosis* isolates confirmed the compensatory role of *ahpC* mutations^25^. *ahpC* mutations did not meet the criteria for being included in the 2021 WHO catalogue of resistance-conferring mutations because they were either too rare or had a low positive predictive value for isoniazid resistance as an independent mutation^23^. However, a study of isoniazid-resistant isolates from Brazil reported that while *ahpC* mutations often co-occurred with *katG* mutations, they were also found in the absence of known resistance mutations in *katG* or *inhA*^26^.

A previous genome-wide survival analysis identified lineages and specific mutations associated with pre-resistance, genomic backgrounds that had a heightened likelihood of acquiring resistance-conferring mutations^27^. Whether *ahpC* acts as in a similar way, generating a “pre-compensated” genomic background, increasing the likelihood of future *katG* mutations, remains unknown.

Our study has several limitations. First, while we sequenced all available *M. tuberculosis* cultures, our final sample size of *M. tuberculosis* genomes was small relative to the number of notified TB cases in our study departments over the study period. Some locally circulating genotypes may, therefore, not be included in our sample and may lead to an underestimate of the contribution of recent transmission to incident TB. However, we sampled over a moderately long timeframe (five years) and included samples from high-incidence prisons and neighboring communities, providing greater opportunity to recover transmission events. Second, surveillance focused on Paraguay’s urban centers, where the majority of TB notifications occur. Future *M. tuberculosis* genomic surveillance in the Chaco, western Paraguay, where incidence is three times higher than in eastern Paraguay^15^, is needed. Additionally, further analysis at the regional-level will be critical for understanding transmission between Paraguay and neighboring countries. Third, we sampled TB infections from prisons at a higher rate than infections outside prisons, potentially biasing upwards estimates of the rate of genomic clustering within prisons compared to outside prisons. Further, we did not have access to more detailed epidemiological data, such as contact information. Future studies integrating genomic data with additional epidemiological data could be used to identify other locations potentially contributing disproportionately to *M. tuberculosis* transmission in Paraguay.

Finally, we sequenced isolates from cultured sputum, as is routinely done for *M. tuberculosis* genomic epidemiology, but which limits the within-host diversity recovered from an individual’s infection. Future research is needed to develop sequencing approaches to recover within-host *M. tuberculosis* variation and incorporate this level of variation into the transmission and ancestral state reconstruction.

Together, our results underscore an urgent need for TB control measures to interrupt ongoing transmission in Paraguay, particularly in high-incidence prison settings, which have an outsized role in broader transmission. Further, the connectivity of Paraguay’s urban centers indicates that TB control needs to be coordinated country-wide.

## Methods

### Study protocol

This research complies with all ethical regulations. The study was approved by the ethics committee of the Central Laboratory of Public Health of the Ministry of Health and Social Welfare of Paraguay (International Certification FWA N° FWAOOO20088) with code CEI-LCSP 91/010217. Informed consent was obtained (Prospective population surveillance).

### Inclusion and ethics

This study was designed and led by a team of researchers in Paraguay (GES, GS, SA) and Brazil (JC). The research seeks to characterize *M. tuberculosis* transmission in order to directly inform priorities of the National Program for Tuberculosis Control (NPTC) of Paraguay (SA, GS) and, more broadly, the Paraguayan Ministry of Health (GS). This work comprises part of the dissertation research of GES and GS. The research was approved by a local ethics review committee and involved minimal risk to study participants.

### Prospective population surveillance

We conducted population-based genomic surveillance in three of Paraguay’s departments: Central, Distrito Capital, and Alto Paraná, which together comprise approximately half (3,392,429 people) of the country’s 2021 population of 7.4 million. Sputum samples are routinely collected from all individuals presenting with symptoms of TB at primary health clinics and sent to the National Program for Tuberculosis Control (NPTC) of Paraguay reference laboratory for microbial diagnostics including culture and smear microscopy. We sequenced all available culture-positive isolates from these three departments.

Study recruitment was done by study staff who visited patients at home and in prisons at the time patients began treatment (Directly Observed Therapy, DOT). At this time, the standard National TB Control Program questionnaire was conducted and patients who chose to enroll provided written consent for sequencing residual mycobacterial cultures for culture-positive samples. Study staff also collected additional demographic, clinical, residential, and epidemiolocal data, including information on history of prior or current incarceration with a structured questionnaire.

Sex was not considered in study design; it was determined through self-report. Of the 488 individual participants, 394 were men and 94 were women, reflecting that recruitment focused on prisons. In 2022, the incarcerated population in Paraguay was 95% male^3^. Median age of study participants was 31 (IQR: 24–44).

### Laboratory and sequencing methods

Sputum samples were cultured in the Ogawa-Kudoh Method^28,29^. Cultures were incubated at 37 °C and observed for growth twice a week for 60 days. *M. tuberculosis* DNA was extracted using Cetyltrimethyl ammonium bromide (CTAB) method^30^.

Sequencing was conducted at the Laboratorio Central de Salud Pública (LCSP), Paraguay Ministry of Health; Centro para el Desarrollo a la investigación Científica (CEDIC), Paraguay*;* and the Translational Genomics Research Institute (TGen), Arizona, US. DNA sequencing libraries were prepared with the Illumina DNA Prep library kit and sequenced on an Illumina MiSeq in Paraguay and an Illumina NextSeq (2 × 151-bp), at TGen. Raw sequence reads for samples passing filters are available at the Sequence Read Archive (PRJNA870648).

### Variant identification

We identified *M. tuberculosis* genomic variation from whole genome sequence data with a pipeline available at https://github.com/ksw9/mtb_pipeline (v1)^31^. We previously conducted a variant identification experiment to compare commonly used mapping and variant calling algorithms in *M. tuberculosis* genomic epidemiology^32^. Briefly, we generated 20 independent Illumina readsets (2 × 151 bp) from the *M. tuberculosis* strain CDC1551 genome in silico, with the next-generation sequence-read simulator ART v. 2.5.8^33^. Measuring performance requires a truth VCF of true variant sites in the query genome with respect to a given reference genome. We generated a truth VCF for the strain CDC1551 query genome with respect to the H37Rv reference genome by pairwise aligning the query genome (strain CDC1551) to *H37Rv* with MUMmer 3.2.0^34^ (*nucmer maxmatch -c 1500*). We identified SNP variants from the pairwise alignments using MUMmer *show-snps*, excluding SNPs with ambiguous mapping and indels (*show-snps -CIr*). Simulated *M. tuberculosis* genomic data and truth VCF files indicating variants with respect to the reference genome are available here: https://purl.stanford.edu/mr554nj9219. We compared variants identified with our pipeline with the “truth VCF” to determine sensitivity and precision of our pipeline (Table S1).

We previously found that the combination of the *bwa*^35^ mapping algorithm and *GATK*^36,37^ variant caller routinely minimizes false positive variant calls with minimal cost to sensitivity as compared to other tool combinations^32^, in particular, when the PE/PPE genes are excluded. We, therefore, used this combination of tools in our pipeline. We report the performance of our computational pipeline in recovering true variants between the CDC1551 query genome and H37Rv reference genome in Table S1.

Briefly, we trimmed low-quality bases (Phred-scaled base quality <20) and removed adapters with Trim Galore v. 0.6.5 (stringency=3)^38^. We used CutAdapt v.4.2 to further filter reads (–nextseq-trim=20–minimum-length=20–pair-filter=any)^39^.To exclude potential contamination, we used Kraken2 to taxonomically classify reads and removed reads that were not assigned to the *Mycobacterium* genus or that were assigned to a *Mycobacterium* species other than *M. tuberculosis*^40^. We mapped reads with bwa v. 0.7.15 (*bwa* mem)^35^ to the H37Rv reference genome (NCBI Accession: NC_000962.3) and removed duplicates with sambamba^41^. We called variants with GATK 4.1 HaplotypeCaller^36^, setting sample ploidy to 1, and GenotypeGVCFs, including non-variant sites in output VCF files. We included variant sites with a minimum depth of 10X and a minimum variant quality score 40 and constructed consensus sequences with bcftools consensus^42^, excluding indels. We excluded SNPs in previously defined repetitive regions (PPE and PE-PGRS genes, phages, insertion sequences and repeats longer than 50 bp)^43^. We identified sub-lineage and evidence of mixed infection with TBProfiler v.4.2.0^44,45^, which is based on the identification of >1000 lineage-specific SNPs. We additionally used TBProfiler with the TBDB repository (https://github.com/jodyphelan/tbdb) which includes >2,000 resistance-associated mutations^44,45^ compiled from several sources, including but not limited to the World Health Organization catalogue^25,44,45^

We do not categorize isolates harboring an independent (*ahpC*) mutation as drug-resistant in phylogenies because it is not considered independently associated with resistance in the World Health Organization Catalogue^23^ or other references.

### Phylogenetic and Bayesian evolutionary analysis

We constructed full-length consensus FASTA sequences from VCF files, setting missing genotypes to missing, and used SNP-sites to extract a multiple alignment of internal variant sites only^46^. We used the R package *ape* to measure pairwise differences between samples (pairwise.deletion=TRUE)^47^. We selected a best fit substitution model with ModelFinder^48^, implemented in IQ-TREE multicore version 2.2.0^49^, evaluating all models that included an ascertainment bias correction for the use of an alignment of SNPs only. The best fit model according to Bayesian Information Criterion was K3Pu + F + ASC + R5, a three substitution types model with unequal base frequencies, an ascertainment bias correction, and a FreeRate model of rate heterogeneity across sites, including four categories. We then fit a maximum likelihood tree with IQ-TREE, with 1000 ultrafast bootstrap replicates^49,50^.

Genomic clustering is often used as a proxy measure of recent *M. tuberculosis* transmission; isolates that are more closely genetically related are hypothesized to be more likely linked through recent transmission rather than travel-associated importation or re-activation of genetically distinct latent infections.^20,51^ We applied a commonly used genetic distance thresholds of 12- and 5- or fewer SNPs to identify genomic clusters^51–53^.

To investigate transmission patterns in the three largest genomic clusters more closely, we fit timed Bayesian trees to multiple sequence alignments with BEAST 2.6.2^54^, using TB notification dates to calibrate tips. Because of the short sampling timeframe of our data, we fixed the substitution rate to 1 × 10^−7^ mutations/site/year, as previously described^55^, and consistent with previous estimates for the *M. tuberculosis* lineage 4 substitution rate^56^. To examine population dynamics in the three largest clusters, we used a Coalescent Bayesian Skyline model^57^ with 5 dimensions, allowing the effective population size to change 4 times over the tree. We additionally fit a Bayesian tree to sublineage 4.2.1.1 samples using a constant population size, fixed substitution rate model. Markov chain Monte Carlo chains were run for 200 million iterations, or longer, if required for convergence, excluding 10% of samples as burn-in. We used *treeannotator* to produce maximum clade credibility trees. We used the R package *beautier* to construct XML files^58^ and corrected XML files for the number of constant positions in SNP alignments. We visualized phylogenetic trees with the R package *ggtree*^59,60^.

We calculated time-scaled haplotype density from a matrix of pairwise SNP distances with the R package *thd* as previously described^22^ and compared time-scaled haplotype density between individuals who were never, formerly, or currently incarcerated with t-tests. We set the *M. tuberculosis* substitution rate to 1 × 10^−7^ substitutions per site per year and included an effective genome length of 3,916,441 basepairs (the length of the reference genome minus the PE/PPE regions excluded from variant calling) and used a short (20 year) and long (50 year) epidemic timescale. We compared time-scaled haplotype density by incarceration status with t-tests and used analysis of variance to test for the independent effect of incarceration status after controlling for *M. tuberculosis* population structure^22^.

We tested for the association between sub-lineage and city with Chi-square tests. We included isolates within the three largest sub-lineages identified (4.1.2.1, 4.3.3, and 4.4.1.1) to avoid comparison of small sample sizes. We conducted discrete ancestral state reconstruction for sampling location with the R package ape for the three largest sub-lineages in our collection^47^. We restricted samples to those from Asunción and Ciudad del Este because of the small sample size outside those cities. We compared symmetric and asymmetric rates models fit with the R package *diversitree* (make.mk2) and compared model fits with analysis of variance^61^. We used stochastic character mapping^62^ in the R package *diversitree*^61^ to sample 500 location histories for each sublineage tree; we summarized these as the number of average movements between cities over the tree.

To test for genomic loci associated with transmissibility, we conducted a bacterial Genome-Wide Association Study implemented in the R package *treewas*^21^. This approach controls for bacterial clonality and population structure by simulating null genomic datasets, in which there is no genotype-phenotype association, to compare with the empirical dataset^21^. We tested if the binary phenotype of membership in a genomic cluster of size 15 or greater (including the three dominant clusters identified in our study) was associated with genotype. We additionally tested for a genotypic association with membership in a genomic cluster of size 10 or more, or any clustering (membership in a genomic cluster of size 2 or more).

### Statistics & reproducibility

We included all *M. tuberculosis* genomes passing coverage and quality thresholds. We excluded isolates with evidence of mixed lineage infection from the analysis. No statistical method was used to predetermine the sample size.

### Reporting summary

Further information on research design is available in the Nature Portfolio Reporting Summary linked to this article.

## Supplementary information

Supplementary Information

Reporting Summary

## Data Availability

Raw sequence data generated in this study have been deposited in the Sequence Read Archive under accession PRJNA870648. The H37Rv reference genome is available on NCBI under accession NC_000962.3) The phylogenetic trees and results of ancestral state reconstruction and all other information displayed in figures are provided in the Source Data files. Source data are provided with this paper.

## Source data

Source Data

## Code availability

Our *M. tuberculosis* variant identification pipeline is available at https://github.com/ksw9/mtb_pipeline^31^.

## References

[1] The World Health Organization. *Global Tuberculosis Report 2021*. https://www.who.int/publications/digital/global-tuberculosis-report-2021 (2021).

[2] World Bank: Poverty and Inequality Platform (Institution/Organization). *Poverty headcount ratio at national poverty lines (% of population) - Paraguay*. (2022).

[3] Institute for Criminal Policy Research. *World Prison Brief*. http://www.prisonstudies.org/ (2019).

[4] Walter KS (2021). The escalating tuberculosis crisis in central and South American prisons. Lancet.

[5] Cords O (2021). Incidence and prevalence of tuberculosis in incarcerated populations: a systematic review and meta-analysis. Lancet Public Heal..

[6] Sequera VG (2020). Increased incarceration rates drive growing tuberculosis burden in prisons and jeopardize overall tuberculosis control in Paraguay. Sci. Rep. 2020 101.

[7] Organización Panamericana de la Salud Programa Regional de Tuberculosis (Institution/Organisation). *VI Reunión Regional: Avances y desafíos del control de la TB en Poblaciones Privadas de Libertad (PPL)* (2013).

[8] Churchyard G (2017). What We Know about Tuberculosis Transmission: An Overview. J. Infect. Dis..

[9] Stucki D (2016). Mycobacterium tuberculosis lineage 4 comprises globally distributed and geographically restricted sublineages. Nat. Genet..

[10] Brynildsrud OB (2018). Global expansion of Mycobacterium tuberculosis lineage 4 shaped by colonial migration and local adaptation. Sci. Adv..

[11] Guerra-Assunção J (2015). Large-scale whole genome sequencing of M. tuberculosis provides insights into transmission in a high prevalence area. Elife.

[12] Ektefaie, Y., Dixit, A., Freschi, L. & Farhat, M. R. Globally diverse Mycobacterium tuberculosis resistance acquisition: a retrospective geographical and temporal analysis of whole genome sequences. *The Lancet Microbe*10.1016/S2666-5247(20)30195-6 (2021). 10.1016/s2666-5247(20)30195-6 PMC8078851 33912853

[13] Gygli SM (2021). Prisons as ecological drivers of fitness-compensated multidrug-resistant Mycobacterium tuberculosis. Nat. Med..

[14] Walter KS (2022). The role of prisons in disseminating tuberculosis in Brazil: A genomic epidemiology study. Lancet Reg. Heal. - Am..

[15] Candia N (2007). First insight into Mycobacterium tuberculosis genetic diversity in Paraguay. BMC Microbiol..

[16] Woodman, M., Haeusler, I. L. & Grandjean, L. Tuberculosis genetic epidemiology: A latin american perspective. *Genes (Basel)*. **10**, (2019). 10.3390/genes10010053 PMC6356704 30654542

[17] Atima, F. *et al*. Prevalence of multidrug-resistant tuberculosis in Latin America and the Caribbean: a systematic review and meta-analysis. 10.1111/tmi.13453 (2020). 10.1111/tmi.13453 32506718

[18] Dean AS (2020). Prevalence and genetic profiles of isoniazid resistance in tuberculosis patients: A multicountry analysis of cross-sectional data. PLOS Med..

[19] Sulisid, G. & Paiid, M. Isoniazid-resistant tuberculosis: A problem we can no longer ignore. 10.1371/journal.pmed.1003023 (2020). 10.1371/journal.pmed.1003023 PMC6974036 31961857

[20] Borgdorff MW, Van Soolingen D (2013). The re-emergence of tuberculosis: what have we learnt from molecular epidemiology?. Clin. Microbiol. Infect..

[21] Collins C, Didelot X (2018). A phylogenetic method to perform genome-wide association studies in microbes that accounts for population structure and recombination. PLOS Comput. Biol..

[22] Rasigade JP (2017). Strain-specific estimation of epidemic success provides insights into the transmission dynamics of tuberculosis. Sci. Rep. 2017 71.

[23] World Health Organization. *Catalogue of mutations in Mycobacterium tuberculosis complex and their association with drug resistance*. https://www.who.int/publications/i/item/9789240028173 (2021).

[24] Sherman DR (1996). Compensatory ahpC gene expression in isoniazid-resistant mycobacterium tuberculosis. Sci. (80-.)..

[25] Coll F (2018). Genome-wide analysis of multi- and extensively drug-resistant Mycobacterium tuberculosis. Nat. Genet..

[26] Silva MSN (2003). Mutations in katG, inhA, and ahpC Genes of Brazilian Isoniazid-Resistant Isolates of Mycobacterium tuberculosis. J. Clin. Microbiol..

[27] Torres Ortiz, A. et al. Genomic signatures of pre-resistance in Mycobacterium tuberculosis. *Nat. Commun*. **12**, 7312 (2021). 10.1038/s41467-021-27616-7 PMC8674244 34911948

[28] Kudoh S, Kudoh T (1974). A simple technique for culturing tubercle bacilli. Bull. World Health Organ..

[29] Palaci M (2013). Contribution of the Ogawa-Kudoh swab culture method to the diagnosis of pulmonary tuberculosis in Brazil. Int. J. Tuberc. Lung Dis..

[30] Schiebelhut, L. M., Abboud, S. S., Omez Daglio, L. E. G. & Swift, H. F. A comparison of DNA extraction methods for high-throughput DNA analyses. 10.1111/1755-0998.12620 (2016). 10.1111/1755-0998.12620 27768245

[31] Walter, K. S. mtb-call pipeline: Phylogeography and transmission of M. tuberculosis spanning prisons and surrounding communities in Paraguay 10.5281/zenodo.7470555 (2022).

[32] Walter KS (2020). Genomic variant-identification methods may alter mycobacterium tuberculosis transmission inferences. Microb. Genomics.

[33] Huang W, Li L, Myers JR, Marth GT (2012). ART: A next-generation sequencing read simulator. Bioinformatics.

[34] Kurtz S (2004). Versatile and open software for comparing large genomes. Genome Biol..

[35] Li H, Durbin R (2009). Fast and accurate short read alignment with Burrows-Wheeler transform. Bioinformatics.

[36] Van der Auwera, G. A. & O’Connor, B. *Genomics in the cloud: using Docker, GATK, and WDL in Terra*. *Genomics in the cloud: using Docker, GATK, and WDL in Terra* (O’Reilly Media, 2020).

[37] Van der Auwera, G. A. et al. From FastQ Data to High-Confidence Variant Calls: The Genome Analysis Toolkit Best Practices Pipeline. in *Current Protocols in Bioinformatics* vol. 43 11.10.1-11.10.33 (John Wiley & Sons, Inc., 2013). 10.1002/0471250953.bi1110s43 PMC4243306 25431634

[38] Krueger, F. Trim Galore. (2019).

[39] Martin, M. Cutadapt removes adapter sequences from high-throughput sequencing reads. *EMBnet.journal* **17**, 10.14806/ej.17.1.200 (2011).

[40] Wood DE, Salzberg SL (2014). Kraken: Ultrafast metagenomic sequence classification using exact alignments. Genome Biol..

[41] Tarasov A, Vilella AJ, Cuppen E, Nijman IJ, Prins P (2015). Sambamba: fast processing of NGS alignment formats. Bioinformatics.

[42] Danecek P (2021). Twelve years of SAMtools and BCFtools. Gigascience.

[43] Brites D (2018). A new phylogenetic framework for the animal-adapted mycobacterium tuberculosis complex. Front. Microbiol..

[44] Phelan JE (2019). Integrating informatics tools and portable sequencing technology for rapid detection of resistance to anti-tuberculous drugs. Genome Med..

[45] Coll, F. et al. Rapid determination of anti-tuberculosis drug resistance from whole-genome sequences. *Genome Med*. **7**, 51 (2015). 10.1186/s13073-015-0164-0 PMC4446134 26019726

[46] Page AJ (2016). SNP-sites: rapid efficient extraction of SNPs from multi-FASTA alignments. Microb. Genomics.

[47] Paradis E, Schliep K (2019). Ape 5.0: An environment for modern phylogenetics and evolutionary analyses in R. Bioinformatics.

[48] Kalyaanamoorthy, S., Minh, B. Q., Wong, T. K. F., Von Haeseler, A. & Jermiin, L. S. modelfinder: fast model selection for accurate phylogenetic estimates. **14**, 587–589 (2017). 10.1038/nmeth.4285 PMC5453245 28481363

[49] Minh BQ (2020). IQ-TREE 2: new models and efficient methods for phylogenetic inference in the genomic era. Mol. Biol. Evol..

[50] Hoang DT, Chernomor O, Von Haeseler A, Minh BQ, Vinh LS (2018). UFBoot2: improving the ultrafast bootstrap approximation. Mol. Biol. Evol..

[51] Walker TM (2013). Whole-genome sequencing to delineate Mycobacterium tuberculosis outbreaks: A retrospective observational study. Lancet Infect. Dis..

[52] Walker TM (2014). Assessment of mycobacterium tuberculosis transmission in oxfordshire, uk, 2007-12, with whole pathogen genome sequences: an observational study. Lancet Respir. Med..

[53] Cervera, B. S. et al. Fine- grain population structure and transmission patterns of Mycobacterium tuberculosis in southern Mozambique, a high TB/ HIV burden area. *Microb. Genomics***accepted**, 844 (2022). 10.1099/mgen.0.000844 PMC9455694 35787782

[54] Bouckaert R (2019). BEAST 2.5: An advanced software platform for Bayesian evolutionary analysis. PLoS Comput. Biol..

[55] Yang C (2022). Phylogeography and transmission of M. tuberculosis in Moldova: a prospective genomic analysis. PLOS Med..

[56] Menardo, F., Duchêne, S., Brites, D. & Gagneux, S. The molecular clock of mycobacterium tuberculosis. *PLoS Pathog*. **15**, e1008067 (2019). 10.1371/journal.ppat.1008067 PMC6759198 31513651

[57] Drummond AJ, Rambaut A, Shapiro B, Pybus OG (2005). Bayesian coalescent inference of past population dynamics from molecular sequences. Mol. Biol. Evol..

[58] Bilderbeek RJC, Etienne RS (2018). babette: BEAUti 2, BEAST2 and Tracer for R. Methods Ecol. Evol..

[59] Yu G, Lam TTY, Zhu H, Guan Y (2018). Two methods for mapping and visualizing associated data on phylogeny using ggtree. Mol. Biol. Evol..

[60] Yu G (2020). Using ggtree to visualize data on tree-like structures. Curr. Protoc. Bioinforma..

[61] Fitzjohn RG (2012). Diversitree: comparative phylogenetic analyses of diversification in R. Methods Ecol. Evol..

[62] Huelsenbeck JP, Nielsen R, Bollback JP (2003). Stochastic mapping of morphological characters. Syst. Biol..

