# Supplementary material for: Phylogeography and transmission of Mycobacterium tuberculosis spanning prisons and surrounding communities in Paraguay
Source: Nat Commun. 2023 Jan 19;14:303. doi: 10.1038/s41467-023-35813-9 (PMC9849832; doi:10.1038/s41467-023-35813-9)
Supplement: Supplementary file 1 — Supplementary Information [file 41467_2023_35813_MOESM1_ESM.docx]

**Supplementary Information**


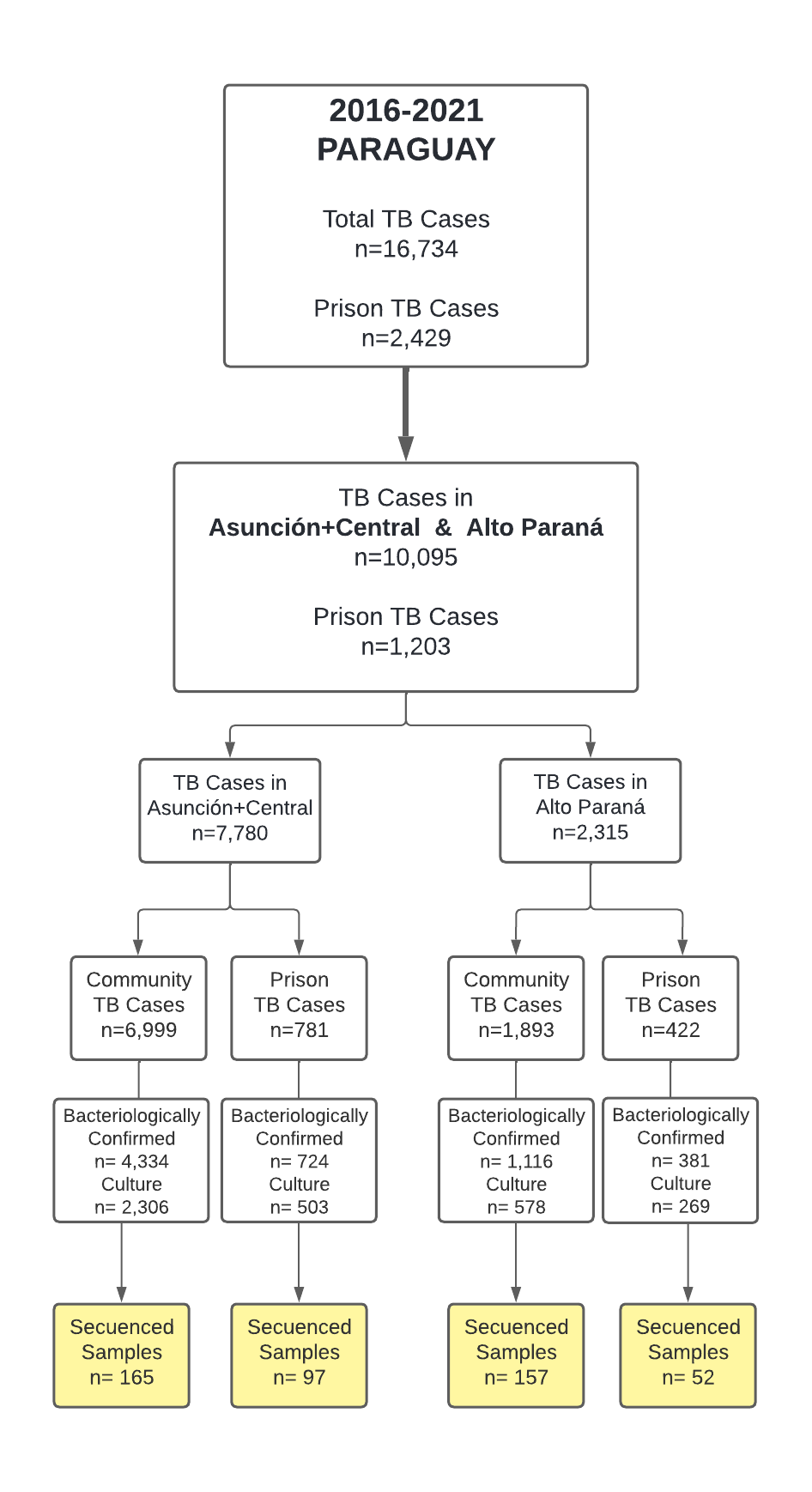


**Figure S1. Genomic *M. tuberculosis* surveillance in Paraguay.** Flowchart indicates the total number of notified cases of TB in Paraguay, from 2016 to 2021; TB cases in the three major urban departments of Paraguay; TB cases in urban departments stratified by incarceration status at the time of TB notification; bacteriologically and culture-confirmed TB cases; and number of cases for which sequenced *M. tuberculosis* passed all quality filters and represented single infections.

**Figure S2. Longitudinal changes in sampled *M. tuberculosis* genomic diversity.** We compared genomic diversity in our study (2016-2021) with that sampled in the only previous genetic study of *M. tuberculosis* in Paraguay^1^. Stacked bar plots indicate the proportion of samples falling in each clade. From left to right, panels indicate the total diversity sampled, samples from Asunción, Ciudad del Este, and from the 2003 study. Panels from the current study are stratified by incarceration status at the time of TB notification; the 2003 study did not present data stratified by incarceration status.

**Figure S3. Time-scaled haplotype density by incarceration status at the time of TB notification.** We measured time-scaled haplotype density, a measure of the centrality of a single tip isolate to all other isolates on the tree, a proxy for recent transmission that considers not only the nearest phylogenetic neighbor, but all tree trips. We calculated time-scaled haplotype density from a matrix of pairwise SNP distances with the R package *thd* as previously described^2^. We set the *M. tuberculosis* substitution rate to 1x10^-7^ substitutions per site per year and included an effective genome length of 3,916,441 basepairs (the length of the reference genome minus the PE/PPE regions excluded from variant calling) and used short (10 year) and long (20 year) epidemic timescales. Boxplots are colored by incarceration status at the time of TB notification and include isolates from n=31 formerly incarcerated individuals, n=149 currently incarcerated, and n=291 never incarcerated individuals. Boxes indicate the interquartile range, lines indicate median values, and whiskers indicate the range of the data. Points outside the whiskers indicate outliers.

**Table S1. Performance metrics.** Performance benchmarking of our pipeline in recovering single nucleotide polymorphisms (SNPs) in simulated Illumina sequence data. For each Metric, the number of simulated sequence sets (Replicates), the mean, median, and standard deviation. True variants indicates the number of “true” SNP variants identified through pairwise alignment of the query genome (strain CDC1551) to H37Rv with MUMmer^3^ (*nucmer maxmatch -c 1500*). We identified SNP variants from the pairwise alignments using MUMmer *show-snps*, excluding SNPs with ambiguous mapping and indels (*show-snps -CIr*). True positives and false positives indicate the number of true or false positive SNPs, respectively, identified by our algorithm. Region indicates whether the PE/PPE genes were excluded from analysis (No PE/PPE) or whether the statistic refers to the full-length M. tuberculosis genome (Genome). In the analysis presented in this paper, we excluded the PE/PPE genes in order to maximize precision.

| Metric | Replicates | Mean | Median | Std. Dev. | Region |
| --- | --- | --- | --- | --- | --- |
| True variants | 20 | 924.00 | 924.00 | 0.00 | No PE/PPE |
| True positives | 20 | 845.40 | 845.00 | 1.14 | No PE/PPE |
| Sensitivity | 20 | 0.92 | 0.92 | 0.00 | No PE/PPE |
| False positives | 20 | 3.35 | 3.00 | 0.49 | No PE/PPE |
| Precision | 20 | 1.00 | 1.00 | 0.00 | No PE/PPE |
| True variants | 20 | 1501.00 | 1501.00 | 0.00 | Genome |
| True positives | 20 | 1148.80 | 1149.50 | 5.24 | Genome |
| Sensitivity | 20 | 0.77 | 0.77 | 0.00 | Genome |
| False positives | 20 | 70.75 | 70.00 | 8.91 | Genome |
| Precision | 20 | 0.94 | 0.94 | 0.01 | Genome |
